# Supplementary material for: Non-enzymatic primer extension with strand displacement
Source: eLife. 2019 Nov 8;8:e51888. doi: 10.7554/eLife.51888 (PMC6872209; doi:10.7554/eLife.51888)
Supplement: Figure 2—source data 1. [file elife-51888-fig2-data1.docx]

**Figure 2 – Source Data 1**

|  | invader (μM) | *k*_obs_ (h^-1^) | | | | | |
| --- | --- | --- | --- | --- | --- | --- | --- |
|  |  | exp 1 | exp 2 | exp 3 | exp 4 | Average | S.D. |
| Octamer  Invader  Room  Temp. | 2 | 0.26 | 0.19 | 0.36 | 0.32 | 0.28 | 0.07 |
|  | 2.8 | 0.54 | 0.30 | 0.51 | 0.59 | 0.48 | 0.13 |
|  | 3.5 | 0.64 | 0.59 | 0.69 | 0.71 | 0.66 | 0.06 |
|  | 5 | 0.96 | 0.96 | 0.80 | 0.77 | 0.87 | 0.10 |
|  | 15 | 0.99 | 1.03 | 0.78 | 0.80 | 0.90 | 0.13 |
|  | 30 | 1.02 | 1.04 | 0.82 | 0.83 | 0.93 | 0.12 |
| Octamer Invader On Ice | 2 | 0.04 | 0.15 | 0.15 | 0.16 | 0.12 | 0.06 |
|  | 2.8 | 0.31 | 0.20 | 0.29 | 0.27 | 0.27 | 0.05 |
|  | 3.5 | 0.31 | 0.36 | 0.38 |  | 0.35 | 0.03 |
|  | 5 | 0.37 | 0.40 | 0.40 | 0.41 | 0.40 | 0.02 |
|  | 15 | 0.40 | 0.39 | 0.41 | 0.43 | 0.41 | 0.02 |
|  | 30 | 0.39 | 0.41 | 0.42 | 0.43 | 0.41 | 0.02 |
| Hexamer Invader Room  Temp. | 7 | N/A | N/A | N/A | N/A | N/A | N/A |
|  | 15 | N/A | N/A | N/A | N/A | N/A | N/A |
|  | 35 | 0.02 | 0.03 | 0.04 | 0.04 | 0.03 | 0.01 |
|  | 70 | 0.05 | 0.04 | 0.10 | 0.07 | 0.07 | 0.02 |
|  | 100 | 0.08 | 0.10 | 0.15 | 0.09 | 0.11 | 0.03 |
| Hexamer Invader On Ice | 7 | 0.10 | 0.08 | 0.11 | 0.10 | 0.10 | 0.01 |
|  | 15 | 0.21 | 0.18 | 0.21 | 0.16 | 0.19 | 0.02 |
|  | 35 | 0.34 | 0.30 | 0.31 | 0.31 | 0.32 | 0.02 |
|  | 70 | 0.40 | 0.40 | 0.35 | 0.36 | 0.38 | 0.03 |
|  | 100 | 0.41 | 0.44 | 0.37 | 0.37 | 0.40 | 0.03 |
