# Supplementary material for: Non-enzymatic primer extension with strand displacement
Source: eLife. 2019 Nov 8;8:e51888. doi: 10.7554/eLife.51888 (PMC6872209; doi:10.7554/eLife.51888)
Supplement: Figure 3—source data 1. [file elife-51888-fig3-data1.docx]

**Figure 3 – Source Data 1**

|  | Mg^2+^ (mM) | *k*_obs_ (h^-1^) | | | | | |
| --- | --- | --- | --- | --- | --- | --- | --- |
|  |  | exp 1 | exp 2 | exp 3 | exp 4 | Average | S.D. |
| Room Temp. | 5 | 0.12 | 0.12 | 0.13 |  | 0.12 | 0.01 |
|  | 20 | 0.53 | 0.48 | 0.47 |  | 0.49 | 0.03 |
|  | 50 | 0.96 | 0.96 | 0.80 | 0.77 | 0.87 | 0.10 |
|  | 100 | 1.75 | 1.63 | 1.66 |  | 1.68 | 0.06 |
|  | 200 | 2.31 | 2.08 | 2.23 |  | 2.21 | 0.12 |
|  | 400 | 2.95 | 2.85 | 2.99 |  | 2.93 | 0.07 |
| On Ice | 5 | N/A | N/A | N/A | N/A | N/A | N/A |
|  | 20 | 0.13 | 0.13 | 0.11 |  | 0.12 | 0.01 |
|  | 50 | 0.37 | 0.40 | 0.40 | 0.41 | 0.40 | 0.02 |
|  | 100 | 0.56 | 0.48 | 0.36 |  | 0.47 | 0.10 |
|  | 200 | 0.75 | 0.74 | 0.74 |  | 0.74 | 0.01 |
|  | 400 | 1.29 | 1.38 | 1.49 |  | 1.39 | 0.10 |
