# Supplementary material for: Non-enzymatic primer extension with strand displacement
Source: eLife. 2019 Nov 8;8:e51888. doi: 10.7554/eLife.51888 (PMC6872209; doi:10.7554/eLife.51888)
Supplement: Figure 4—source data 1. [file elife-51888-fig4-data1.docx]

**Figure 4 – Source Data 1**

|  | C*C (mM) | *k*_obs_ (h^-1^) | | | | | |
| --- | --- | --- | --- | --- | --- | --- | --- |
|  |  | exp 1 | exp 2 | exp 3 | exp 4 | Average | S.D. |
| Case **(1)** | 0.1 | 2.06 | 2.12 | 2.03 | 2.17 | 2.09 | 0.06 |
|  | 0.2 | 3.39 | 3.36 | 3.37 | 3.72 | 3.46 | 0.17 |
|  | 0.5 | 5.63 | 5.77 | 5.62 | 6.12 | 5.79 | 0.23 |
|  | 1 | 7.01 | 7.85 | 7.02 | 7.41 | 7.32 | 0.40 |
|  | 2 | 8.19 | 8.16 | 8.43 | 8.16 | 8.24 | 0.13 |
|  | 5 | 8.94 | 8.77 | 8.95 | 9.18 | 8.96 | 0.17 |
|  | 10 | 9.08 | 8.95 | 9.30 | 9.70 | 9.26 | 0.33 |
|  | 20 | 9.89 | 9.13 | 9.76 | 9.32 | 9.52 | 0.36 |
| Case **(2)** | 0.1 | 0.40 | 0.35 | 0.34 | 0.36 | 0.36 | 0.03 |
|  | 0.2 | 0.77 | 0.77 | 0.79 | 0.75 | 0.77 | 0.02 |
|  | 0.5 | 1.75 | 1.84 | 1.86 | 1.86 | 1.83 | 0.05 |
|  | 1 | 2.93 | 3.08 | 2.99 | 3.19 | 3.05 | 0.11 |
|  | 2 | 4.26 | 4.50 | 4.77 | 4.62 | 4.54 | 0.22 |
|  | 5 | 6.20 | 6.60 | 6.56 | 6.54 | 6.48 | 0.18 |
|  | 10 | 8.19 | 7.90 | 7.79 | 7.71 | 7.90 | 0.21 |
|  | 20 | 8.80 | 8.57 | 8.54 | 8.48 | 8.60 | 0.14 |
| Case **(3)** | 0.1 | N/A | N/A | N/A | N/A | N/A | N/A |
|  | 0.2 | 0.06 | 0.06 | 0.06 | 0.06 | 0.06 | 0.00 |
|  | 0.5 | 0.18 | 0.21 | 0.19 | 0.20 | 0.19 | 0.02 |
|  | 1 | 0.36 | 0.46 | 0.40 | 0.39 | 0.40 | 0.04 |
|  | 2 | 0.68 | 0.87 | 0.76 | 0.79 | 0.77 | 0.08 |
|  | 5 | 1.37 | 1.44 | 1.49 | 1.53 | 1.46 | 0.07 |
|  | 10 | 1.91 | 2.07 | 2.09 | 2.27 | 2.08 | 0.15 |
|  | 20 | 2.44 | 2.52 | 2.62 | 2.80 | 2.59 | 0.16 |
