# Supplementary material for: Non-enzymatic primer extension with strand displacement
Source: eLife. 2019 Nov 8;8:e51888. doi: 10.7554/eLife.51888 (PMC6872209; doi:10.7554/eLife.51888)
Supplement: Figure 6—source data 1. [file elife-51888-fig6-data1.docx]

**Figure 6 – Source Data 1**

|  | Time (h) | Fluorescence (A.U.) | | | | |
| --- | --- | --- | --- | --- | --- | --- |
|  |  | exp 1 | exp 2 | exp 3 | Average | S.D. |
| no bound blocker | 0 | 1788.3 | 1528.4 | 1584.7 | 1633.8 | 136.7 |
|  | 1 | 2000.0 | 1765.7 | 1650.8 | 1805.5 | 177.9 |
|  | 3 | 2059.3 | 1862.8 | 1790.6 | 1904.2 | 139.0 |
|  | 5 | 2052.6 | 1875.4 | 1843.6 | 1923.9 | 112.6 |
|  | 7 | 2138.1 | 1966.6 | 1947.1 | 2017.3 | 105.1 |
|  | 24 | 2176.7 | 1994.4 | 1986.3 | 2052.5 | 107.7 |
|  | 48 | 2165.7 | 1984.7 | 1930.0 | 2026.8 | 123.4 |
| strand displacement reaction | 0 | 13.8 | 14.3 | 14.2 | 14.1 | 0.3 |
|  | 1 | 37.3 | 38.6 | 39.3 | 38.4 | 1.0 |
|  | 3 | 108.3 | 132.5 | 127.8 | 122.9 | 12.8 |
|  | 5 | 207.8 | 254.9 | 242.0 | 234.9 | 24.3 |
|  | 7 | 328.7 | 402.7 | 371.4 | 367.6 | 37.2 |
|  | 24 | 885.4 | 972.2 | 918.9 | 925.5 | 43.7 |
|  | 48 | 1187.7 | 1196.8 | 1134.9 | 1173.1 | 33.4 |
| no monomers | 0 | 12.26 | 12.37 | 13.18 | 12.6 | 0.5 |
|  | 1 | 23.45 | 23.08 | 23.47 | 23.3 | 0.2 |
|  | 3 | 30.10 | 28.20 | 29.59 | 29.3 | 1.0 |
|  | 5 | 31.47 | 29.52 | 30.81 | 30.6 | 1.0 |
|  | 7 | 31.92 | 29.74 | 31.11 | 30.9 | 1.1 |
|  | 24 | 32.56 | 30.54 | 31.67 | 31.6 | 1.0 |
|  | 48 | 35.17 | 32.85 | 33.74 | 33.9 | 1.2 |
| no invaders | 0 | 3.91 | 4.52 | 4.13 | 4.2 | 0.3 |
|  | 1 | 4.68 | 4.99 | 5.01 | 4.9 | 0.2 |
|  | 3 | 5.33 | 6.07 | 6.16 | 5.9 | 0.5 |
|  | 5 | 6.06 | 7.04 | 6.77 | 6.6 | 0.5 |
|  | 7 | 6.95 | 8.22 | 7.30 | 7.5 | 0.7 |
|  | 24 | 15.03 | 16.27 | 12.89 | 14.7 | 1.7 |
|  | 48 | 25.92 | 27.20 | 22.25 | 25.1 | 2.6 |
| no monomers, no invaders | 0 | 2.88 | 3.50 | 3.41 | 3.3 | 0.3 |
|  | 1 | 2.97 | 3.05 | 3.10 | 3.0 | 0.1 |
|  | 3 | 2.99 | 3.37 | 3.17 | 3.2 | 0.2 |
|  | 5 | 3.18 | 3.60 | 3.42 | 3.4 | 0.2 |
|  | 7 | 3.42 | 3.80 | 3.68 | 3.6 | 0.2 |
|  | 24 | 4.42 | 4.90 | 4.69 | 4.7 | 0.2 |
|  | 48 | 5.84 | 6.55 | 6.34 | 6.2 | 0.4 |
